# A Study of Bite Force and Various Variables in Children Segregated by Angle's Classification

**DOI:** 10.5005/jp-journals-10005-1148

**Published:** 2012-08-08

**Authors:** Sarabjeet Singh, Navreet Sandhu, Rita Kashyap

**Affiliations:** Professor and Head, Department of Orthodontics, Bhojia Dental College and Hospital, Solan, Himachal Pradesh, India e-mail: sarabjeet3400@yahoo.co.in; Reader, Department of Prosthodontics, National Dental College and Hospital, Patiala, Punjab, India; Senior Lecturer, Department of Orthodontics, Bhojia Dental College and Hospital, Solan, Himachal Pradesh, India

**Keywords:** Bite force, Angle's classification

## Abstract

The stomatographic system has been studied by several researchers, yet it is still unclear, weather a genetically determined facial morphology decides the strength of masticatory muscles,^1^ or weather a strong musculature influences the form of the face. This formed the basis of present study to relate muscle activity with various malocclusions. Thus, 60 samples of younger age group were divided according to Angle classification and maximum bite force was recorded among the groups. Newly designed bite force recorder was used for recording bite force at molar and at incisal region. Influence of various independent variables like gender, overjet and overbite of the subjects on the bite force was also checked. It was concluded that maximum bite force at intercuspal position (molar) and anterior bite position (incisal) were not significantly different between normal, class I, class II div 1 and class III malocclusion groups. There was no significant correlation between incisal bite force and overjet or overbite, but there was a highly significant difference (p < 0.001) between the males and females for maximum bite force at intercuspal position, with males biting harder than the females.

**How to cite this article:** Singh S, Sandhu N, Kashyap R. A Study of Bite Force and Various Variables in Children Segregated by Angle's Classification. Int J Clin Pediatr Dent 2012;5(2):118-123.

## INTRODUCTION

The craniomandibular function is determined by the complex and interrelated components comprising the morphology and biomechanics of the muscles, joints, teeth and the neuromuscular system. The relationship between form and function of the stomatographic system has been studied by several researchers and it is still not clear, whether a genetically determined facial morphology decides the strength of masticatory muscles,^1^ or weather a strong musculature influences the form of the face.^2,3^

Several clinical and animal experimental studies have shown the significant role played by the masticatory muscle function in craniofacial growth. To evaluate clinically the physiologic characteristics of the masticatory muscles, various methods like measurement of myoelectric activity,^4,5^ bite force^6-8^ have been used. It has been shown that relatively large forces are generated when teeth are brought into occlusion and these forces decrease when the bite point is moved anteriorly.^9^ There is a controversial relationship between bite force and age and sex of patients. In some investigations,^7^ no difference between gender was detected, whereas in others,^3,10^ males produced greater bite force than the females. Bite force has been shown to increase with age till a specific age and then the levels start decreasing, but the cut off age for this change is still not known. The variability of the results of bite force has often been considerable with a large number of factors influencing the values obtained.^10^

Angle's classification was used for differentiating the malocclusion groups. There is a reported difference in the muscle activity of subjects with normal occlusion and with various malocclusions,^4^ between children and adults and also between males and females. Much attention has been paid to the study of maximum bite force and masticatory muscle activity in subjects with advanced occlusal wear but evaluation of general muscle strength in such subjects has received a scant attention. These studies were usually limited to involving either a specific muscle, a specific malocclusion or a specific position, but limited in relating them all together. Thus, this study was conducted with the following aims and objectives in mind:

1. To measure the maximum bite force in younger subjects with normal occlusion and with various malocclusions segregated by Angle's classification.
2. To check for the influence of various independent variables like gender, overjet and overbite of the subjects on the bite force.

## MATERIALS AND METHODS

### Sample

The study comprised of 60 subjects who were divided into four groups as follows:

Group A consisted of 15 subjects with normal occlusion.

Group B consisted of 15 subjects with Angle's class I malocclusion.

Group C consisted of 15 subjects with Angle's class II div 1 malocclusion.

Group D consisted of 15 subjects with Angle's class III malocclusion.

Young adolescents of 12 to 16 years were selected at random from the patients reporting at orthodontic clinic.

### Selection Criteria of Subjects

1. Normal occlusion (group A)
  - Presence of Angle's class I molar relationship.
  - Normal overjet and overbite.
  - No crowding, spacing, rotations, crossbite or open bite.
  - No history of orthodontic treatment.
  - Presence of full complement of teeth.
2. Malocclusion (groups B, C, D)
  - Classification of malocclusion according to molar relationship (Angle's criteria).
  - Type of skeletal pattern (clinical diagnosis).
  - No history of orthodontic treatment.
  - Presence of full complement of teeth.
  - No large restorations/carious lesions on permanent first molars and incisors.
  - No open bite (Anterior and lateral).

### Apparatus

Bite Force Recorder

The bite force recorder^11^ consists of a detailed state of the art apparatus which was carefully selected and individually crafted using technical expertise when required. The actual device was developed in conjunction with the superior technical know how as well as advanced armamentarium at Central Scientific Instruments Organization, Chandigarh and Precision Tools Galaxy, Chandigarh.

**Fig. 1:**
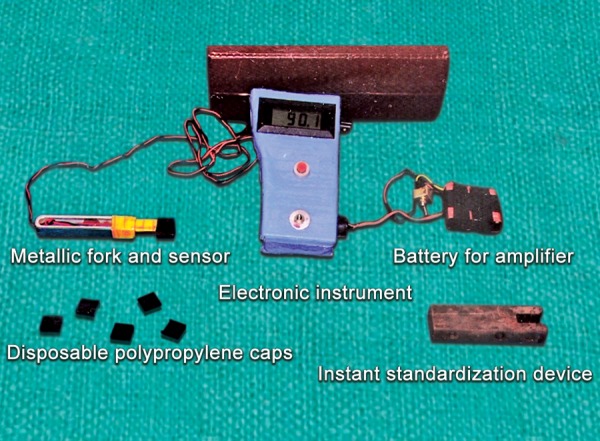
Parts of gnathodynamometer

**Fig. 2:**
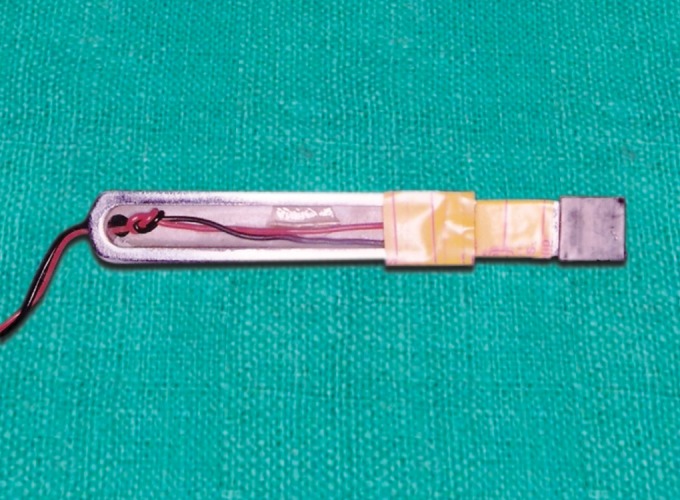
Parts of metallic fork and sensor

It consisted of following components (Fig. 1):

- Metallic fork and sensor (Fig. 2)
- Electronic instrument
- Batteries for instrumentation amplifier, digital panel meter and wheatstone bridge
- Instant standardization device
- Disposable polypropylene caps.

It is more sensitive, accurate, reproducible, compact, battery operated, hygienic due to disposable covers and has the ability to produce accurate readings in a simplified way which is very helpful and suitable for field studies as well as the clinics.

### Taking Records

**Fig. 3:**
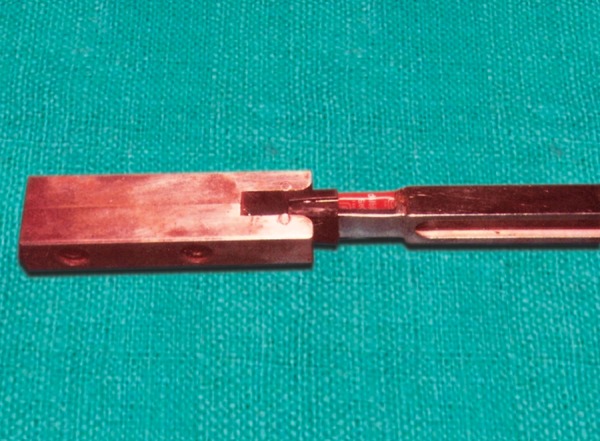
Calibration by instant standardization device before and after each recording

The patients were seated on a dental chair with head unsupported and positioned so that the Frankfort horizontal plane would be parallel to the floor. The patients were explained about the procedure and asked to bite maximally when told. The bite force recorder was calibrated by instant standardization device before and after each recording (Fig. 3).

**Fig. 4:**
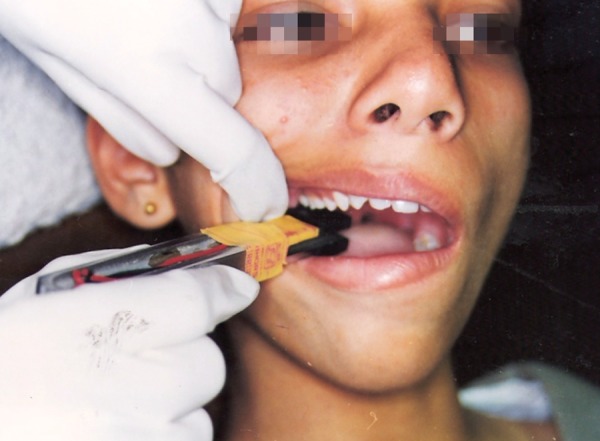
Technique for recordings the maximum bite force (MBFP1)

The fork was placed parallel to the dental arch so that biting end was positioned in the right maxillary first molar region (Fig. 4). A series of three consecutive recordings were taken and noted. A rest period of 1 minute was given between each recording to prevent muscle fatigue. Mean of the three recordings was taken as the maximum bite force (MBF) in the molar region (maximal intercuspal position, MBFP1).

**Fig. 5:**
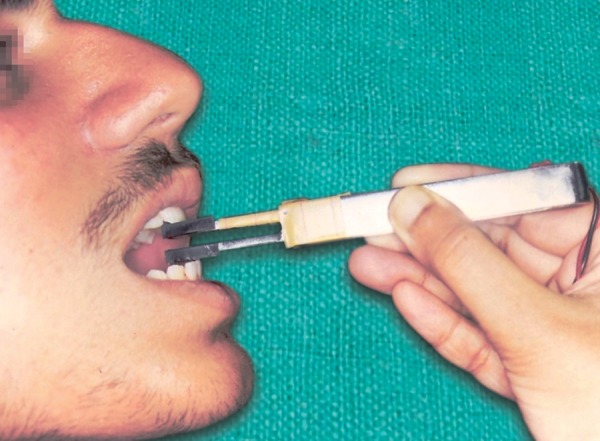
Technique for recordings the maximum bite force (MBFP2)

For the incisal bite position, the bite fork was held parallel to the floor and then was carried to the patient's mouth, so that the marker on the acrylic pad was positioned against the incisal edges of the maxillary incisors (Fig. 5). Each patient was asked to slide the mandible forward, without lateral shift, to establish an end-to-end relationship and bite as hard as one could. Three consecutive recordings were taken and their mean was recorded as the maximum bite force (MBF) at incisal position (interincisal position, MBFP2). No attempt was made to sustain the bite. The plastic caps were changed for every subject.

Studying Independent Variables

The gender, overjet, overbite of all the subjects in four groups were also recorded to check for the influence of various independent variables on the bite force.

### Statistical Analysis

Data were analyzed by conventional statistical methods, i.e. arithmetic mean and standard deviation. Difference between the means for the groups A, B, C and D were tested by analysis of variation (ANOVA) followed by a multiple range test of modified LSD (Bonferroni) at 0.05 level of significance.

Difference between the means for genders for various groups was tested by student's unpaired t-test.

## RESULTS

The study was conducted on 60 subjects, who were divided into four groups, with each group consisting of 15 subjects. These six groups were grossly divided into two major groups, under which they were further studied, that is the younger age group (group A, B, C and D) between 12 to 16 years.

Group A (normal occlusion) consisted of 7 males and 8 females, with the mean age of 14.8 ± 1.47 years, group B (Angle's class I malocclusion) consisted of 6 males and 9 females, with the mean age of 14.4 ± 1.63 years, group C (Angle's class II div 1 malocclusion) consisted of 8 males and 7 females, with the mean age of 13.8 ± 1.26 years, group D (Angle's class 111 malocclusion) consisted of 8 males and 7 females, with the mean age of 14.6 ± 1.29 years (Table 1).

Comparison of maximum bite force and other independent variables in four groups (Table 2).

A comparison of maximum bite force at intercuspal position (MBFP2) and interincisal position (MBFP3) was done for different malocclusion groups (Table 2). The other variables like overbite (O BITE) and overjet (O JET) were also studied for the different groups. A multiple range test of modified LSD (DonfciTuni) was applied to test at 0.05 level of significance.

The mean MBFP2 was 445.48 ± 52.23N for group A; 449.67 ± 66.37N for group B; 457.56 ± 43.99N for group C and 451.20 ± 59.35N for group D. The corresponding values for mean MBFP3 were 120.88 ± 15.91N, 120.12 ± 22.76N, 120.67 ± 28.57N and 108.49 ± 15.41N. The results showed no significant difference at MBFP2 and MBFP3 between normal, class 1, class II div 1 and class III malocclusion groups.

A comparison of overbite and overjet relation between the various malocclusion groups showed a significant difference at 0.05 level. The mean overbite for group A was 17.66 ± 4.16%; for group B was 32.0 ± 15.78%; for group C was 60.66 ± 29.69% and for group D was 11.33 ± 8.33%. There was a significant difference between group A and C; group B and C; group B and D and between group C and D. Subjects with class II div I malocclusion (group C) showed the maximum overbite and those with class 111 showed the minimum.

For comparing the overjet, the mean overjet was 1.76 ± 0.62 mm for group A; 4.43 ± 2.06 mm for group B; 8.46 ± 2.87 mm for group C and 0.83 ± 0.64 mm for group D. There was a significant difference between group A and B; group A and C; group B and C; group II and D and also between group C and D. The mean overjet for class II div I was largest and that for class III was least.

Comparison of maximum bite force and other independent variables between males and females (Table 3).

Among the 60 subjects, there were 29 males and 31 females, who were nearly equally distributed among the four groups (A, B, C and D).

**Table 1:** **Table 1:** Distribution of sample

		*Age range (yrs)*		*Age (yrs) mean, SD*		*Sex*				*Total*	
						*Male*		*Female*			
Group A (normal)		12.0-16.0		14.8 ± 1.47		7		8		15	
Group B (class I)		12.0-16.0		14.4 ± 1.63		6		9		15	
Group C (class II)		12.0-16.0		13.80 ± 1.26		8		7		15	
Group D (class III)		12.0-16.0		14.6 ± 1.29		8		7		15	

**Table 2:** **Table 2:** Comparison of maximum bite force and various independent variables in different malocclusion groups

*Variables*		*Group A (normal)*		*Group B (class I)*		*Group C (class II)*		*Group D (class III)*		Significant* difference	
AGE (yrs) mean ± SD		14.8 ± 1.47		14.4 ± 1.63		13.80 ± 1.26		14.6 ± 1.29		NS	
MBFP2(N) mean ± SD		4452.48 ± 52.23		449.67 ± 66.37		457.56 ± 43.99		451.20 ± 59.35		NS	
MBFP3 (N) mean ± SD		120.88 ± 15.91		120.12 ± 22.76		120.67 ± 28.57		108.49 ± 15.41		NS	
F PRESS (N) mean ± SD		47.78 ± 11.51		48.30 ± 13.37		43.93 + 10.08		46.55 ± 10.00		NS	
O BITE (%) mean ± SD		17.66 ± 4.16		32.0 ± 15.78		60.66 + 29.69		1133 ± 8.33		b,d,e,f	
O JET (mm) mean ± SD		1.76 ± 0.62		4.43 ± 2.06		8.46 ± 2.87		0.83 ± 0.64		a,b,d,e,f	

*Based on analysis of variance at 0.05 level.

a: A *vs* B b**:** A *vs* C c**:** A *vs* D

d: B *vs* C e: B *vs* D f**:** C *vs* D

NS: Not significant; P2: Intercuspal position and P3: Interincisal position

**Table 3:** **Table 3:** Comparison of maximum bite force and various independent varibles between males and females in different malocclusion groups

*Variables*		*Male (n = 29)*		*Female (n = 31)*		*t-value*		p-value^*^	
Age (yrs) mean ± SD		14.68 ± 1.48		14.12 ± 1.31		0.48		NS	
MBFP2 (N) mean ± SD		482.39 ± 43.56		421.59 ± 47.91		5.13		0.0005	
MBFP3 (N) mean ± SD		120.82 ± 21.37		114.47 ± 21.45		1.15		NS	
O BITE (%) mean ± SD		32.41 ± 28.39		28.54 ± 23.02		0.58		NS	
O JET (mm) mean ± SD		3.82 ± 3.54		3.91 ± 3.46		–0.10		NS	

*Based on students unpaired t-test; P2: Intercuspal position and P3: Interincisal position; NS: Not significant

There was no significant difference in age between males and females, with the mean age being I4.68 ± 1.48 years for males and 14.12 ± 1.31 years for females, applying the unpaired t-test showed a highly significant difference (p: 0.0005) between the males and females for maximum bite force at intercuspal position (MBFP2). The mean MBFP2 for males was higher (482.39 ± 43.56N) than females (421.59 ± 47.91N), but there was no significant difference for incisal bite force, with mean MBFP3 being 120.82 ± 2l.37N for males and 114.47 ± 21.45N for females.

The mean overbite for males was 32.41 ± 28.39% and 28.54 ± 23.02% for females and the mean overjet was 3.82 ± 3.54 mm for males and 3.91 ± 3.46 mm for females. There was no significant difference between males and females for overbite and overjet measurements.

## DISCUSSION

The relationship between form and function of the stomatognathic system has been studied by several researchers and it is still not clear, whether a genetically determined facial morphology decides the strength of masticatory muscles, or whether a strong musculature influences the form of the face.

Several clinical and animal experimental studies have shown the significant role played by the masticatory muscle function in craniofacial growth.^1-3^ To evaluate clinically the physiologic characteristics of the masticatory muscles, various methods like measurement of myoelectric activity, bite force recording and endurance test have been used. The most successful of the entire lot of bite force recorders consisted of a metallic fork, an electronic instrument, an instant standardization device and disposable caps. This forms the basis of the framework of the bite force recorder designed entirely in house by us.^11^ It is more sensitive, accurate, reproducible, compact, battery operated, hygienic due to disposable covers and has the ability to produce accurate readings in a simplified way.

There is a reported difference in the muscle activity of subjects with normal occlusion and with various malocclusions,^4^ between children and adults and also between males and females. However, previous studies were usually limited to involving either specific muscle, a specific malocclusion or a specific position. Hence, this study was planned to overcome these limitations and had the aims and objectives to measure the maximum bite force in younger subjects with normal occlusion and with various malocclusions segregated by Angle's classification and also to check for the influence of various independent variables like gender, overjet and overbite of the subjects on the bite force.

The study comprised of 60 subjects who were divided into four groups (group A, B, C and D) between 12 to 16 years. The group A was taken as the control for the remaining three groups in the younger age group and consisted of 15 subjects with normal occlusion (Angle's class 1 molar relation). Group B consisted of subjects with Angle's class I malocclusion, group C with Angle's class II div 1 malocclusion and group D with Angle's class III malocclusion. The Angle's classification was chosen as selection criteria because this enabled to investigate a large number of individuals without using radiographs, thereby fulfilling strict ethical considerations relating to radiation protection. The sample among the young adolescents was divided equally between the genders with 29 males mid 31 females to find a possible difference in maximum bite force care was taken to avoid the subjects with large restorations, carious lesions on permanent first molars and incisors, as there is a reported decrease in bite force in subjects with large restorations.

### Comparison of Maximum Bite Force and Other Independent Variables (Overjet and Overbite) in Four Groups

The maximum bite force at intercuspal position (molar) and anterior bite position (incisal) was not significantly different between normal, class I, class II div 1 and class III malocclusion groups (Table 2). This was in agreement with Kiliarides et al^8^ who showed no difference in bite force between subjects divided by Angle's classification. No unanimity exists between different studies,^4,5^ regarding the function of masticatory muscles and its relation to sagittal deviations of the facial morphology and occlusion of teeth. The mean maximum bite force sample was 450.98 ± 21.47N for intercuspal position and 117.54 ± 2I.47N for interincisal position, which were close to those obtained by previous studies in the similar age groups,^7^ while others^12^ have reported lower values as compared to the present study. Bilt et al^17^ measured bite during bilateral and unilateral maximum clenching as 569N and 430N which are in concordance with present study. Another study reported that bite force did not vary significantly between the Angles malocclusion types maximum bite force increased significantly with age in girls.^18^

In studies on occlusal bite force, the variability of results has often been considerable. These inconsistent results seem to be due to lack of control of certain variables, which are divided into (1) variations due to bite force recorder and (2) intraindividual variables. Variations due to bite force recorder could be due to lack of flexibility of the transducer element, dynamic responsiveness and accuracy of the transducer, vertical separation of the jaws produce due to size of transducer element, location of bile force transducer and distraction of condyles excessively due to the size of the transducer. The reported factors for the intraindividual variations are the variations in jaw morphology, state of dentition, sensitivity of teeth, muscles and temporomandibular joints, degree of physical training and masticatory habits, general health/mental state of person (fear of dental damage)', attitude of the investigator and subject, head posture, different subject populations, location of bite point, histochemical fiber type of jaw muscles, age and sex of the subjects, subjects reluctance to bite maximally, individual differences in muscle strength, geometrical arrangements of the respective jaw muscle lever system and muscle cross- section sizes of the individual.^3,10,12,13,19^

### Comparison of Maximum Bite Force between Males and Females

The difference between males and females regarding maximal bite force has been controversial. In some investigations,^7,8^ no difference between gender was detected, whereas in others^3,10,14,15^ males produced greater bite force than females. In present study, there was a highly significant difference (p < 0.001) between the males and females for maximum bite force at intercuspal position (MBFP3), with males biting harder than the females. The significantly higher level of force in males as compared to that in females in the present study fits well with the findings of Ingervall and Minder^10^ and Corrucini et al^14^ and, who recorded the maximum bite force in a sinylar age group.

For the incisal bite force there was no significant difference in males and females. This was in agreement with Kiliaradis et al^8^ and Waltimo and Kononen,^15^ who showed the difference in maximum bite force for molars only and not for incisors.

The gender difference in bite force may be explained due to the greater muscular potential of males as compared to that of females. Sasaki et al^16^ have noted that jaw muscle size alone was the most important factor in explaining the variations in bite force between the genders. The morphologic variations in facial skeleton between men and women could also explain the difference. Bailit et al^3^ have reported that men's teeth are 1 to 4% larger than those of women and thus have more supportive tissue to tolerate higher value of bite force in the molar region. Pain in teeth has been shown as a major limiting factor for biting harder in the incisal region.^15^ This could be the reason why men are not able to use their ability for greater bite force in the incisal region and account for the observed negligible difference of incisal bite force between the genders.

## CONCLUSION

It has been shown that relatively large forces are generated when teeth are brought into occlusion and these forces decrease when bite point is moved anteriorly. There is a controversial relationship between bite force and age and sex of patients. This study had been an attempt to relate maximum bite force with type of malocclusion and various variables. Maximum bite force at intercuspal position (molar) and anterior bite position (incisal) were not significantly different between normal, class I, class II div 1 and class III malocclusion groups. There was no significant correlation between incisal bite force and overjet or overbite. There was a highly significant difference (p < 0.001) between the males and females for maximum bite force at intercuspal position (MBFP3), with males biting harder than the females. For the incisal bite force there was no significant difference in males and females. Also bite force recorder used was highly sensitive, accurate, reproducible, portable, compact, battery operated, hygienic and which produced a moderate opening of jaws. Although sample size was large enough to draw a conclusion but inclusion of age factor variation and also effects of various muscles of mastication individually would show a better relationship regarding the forces generated within an oral cavity.
